# Cardiogenic shock associated with loco-regional anesthesia rescued with left ventricular assist device implantation

**DOI:** 10.1186/1749-8090-5-126

**Published:** 2010-12-08

**Authors:** Louis E Samuels, Elena Casanova-Ghosh, Christopher Droogan

**Affiliations:** 1 Department of Cardiothoracic Surgery, Lankenau Hospital, Wynnewood, PA, USA; 2 Department of Medicine, Division of Cardiology, Lankenau Hospital, Wynnewood, PA, USA

## Abstract

A healthy 53 year old man developed profound cardiogenic shock following instillation of bupivacaine-lidocaine-epinephrine solution as a locoregional anesthetic for elective outpatient shoulder surgery. Intubation, resuscitation, and transfer to the nearby hospital were done: echocardiography showed profound biventricular dysfunction; cardiac catheterization showed normal coronary arteries. Despite placement of an intra-aortic balloon pump and intravenous vasoactive drugs, the patient remained in shock. Stabilization was achieved with emergent institution of cardiopulmonary bypass and placement of a temporary left ventricular assist device (LVAD). Twenty-four hours later, cardiac function normalized and the LVAD was removed. The patient was discharged five days later and remained with normal heart function in three-year follow-up.

## Introduction

Interscalene nerve blockade for shoulder surgery is a common practice among anesthesiologists and orthopedic surgeons [1]. Although major complications are uncommon, the most life-threatening ones are cardiotoxic in nature [2]. Depending upon which agents are utilized, the effects may be transient and rapidly resolve or prolonged and require advanced resuscitative measures [3]. The case of a 53 year old man who developed acute cardiogenic shock during administration of a loco-regional anesthetic for outpatient elective shoulder surgery is presented. Emergent institution of cardiopulmonary bypass and placement of a temporary left ventricular assist device (LVAD) were necessary as a rescue therapy and bridge to myocardial recovery.

## Clinical Summary

A 53 year old healthy man presented with shoulder pain for outpatient elective arthroscopic surgery. The past medical and surgical histories were hypertension, pancreatitis, gastro-esophageal reflux disease, Lyme disease, and appendectomy. He was a former smoker and drank alcoholic beverages at social occasions. His medications included omeprazole and pantoprazole; he was not allergic to drugs or other products.

At the outpatient surgical center, the patient was placed in the sitting position and prepared for application of an interscalene nerve block. Routine monitoring included three-lead electrocardiography, pulse oximetry, and a blood pressure cuff. The baseline vital signs were the following: Normothermia, BP 129/75 mmHg, PR 61 bpm, and RR 16. A solution of 0.5% Marcaine (Hospira, Inc., Lake Forest, IL) and 1.5% lidocaine with epinephrine (1:200,000) was instilled using a standard technique. Shortly after the instillation, the patient became tachycardic and hypertensive; beta-blockade was given with intravenous labetalol. The patient became hypotensive and demonstrated signs of pulmonary congestion. Intubation was performed and vasoactive drugs were given including norepinephrine and epinephrine. The cardiac rhythm appeared to be ventricular tachycardia which then degenerated into ventricular fibrillation requiring cardiopulmonary resuscitation and electrical defibrillation--advanced cardiac life support (ACLS). The patient was transferred to the nearby hospital where echocardiography showed severe global dysfunction. Emergent cardiac catheterization showed normal coronary anatomy; an intra-aortic balloon pump was placed; high-dose dopamine was added to the norepinephrine infusion without improvement in the shock state. Cardiac surgery was consulted and the patient transported to the operating room in extremis condition.

**Figure 1:**
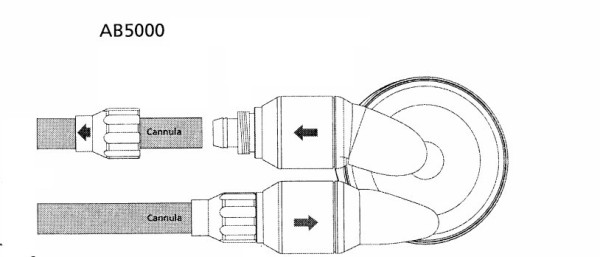
**Abiomed AB5000 VAD™**.

Cardiopulmonary bypass was rapidly instituted following a median sternotomy. The heart appeared markedly distended with severe biventricular failure. An Abiomed AB5000™ (Abiomed Inc., Danvers, MA) left ventricular assist device (LVAD) was placed (Figure 1). Restoration of hemodynamics was immediately observed with subsequent reduction in the vasoactive infusions. The heart, however, remained severely dysfunctional. The sternum was left open in order to avoid compression of the unsupported right ventricle--a VAC™ (Kinetic Concepts, Inc., San Antonio, TX) dressing was applied to protect the LVAD cannulae and provide a sterile barrier to the mediastinum. The patient was transferred to a quaternary care center for further management.

The following day, preparation was made for sternal closure. In the operating room, the VAC™ dressing was removed and the heart inspected. The gross findings confirmed the TEE assessment--biventricular function was restored to normal. The LVAD was weaned successfully and removed. Sternal closure was also accomplished. The remainder of the hospitalization was unremarkable; the patient was discharged on the sixth postoperative day. No further cardiac episodes have occurred in three years follow-up.

## Discussion

Although historically a safe and effective means of anesthesia [4], cardiovascular toxicity from loco-regional anasthetics has been known for over three decades [5]. Rarely, though, is it necessary to institute extreme forms of cardiopulmonary resuscitation, such as cardiopulmonary bypass [6]. However, advanced resuscitation with various agents, intubation, and occasionally defibrillation for arrhythmia have been described [7-10].

**Figure 2:**
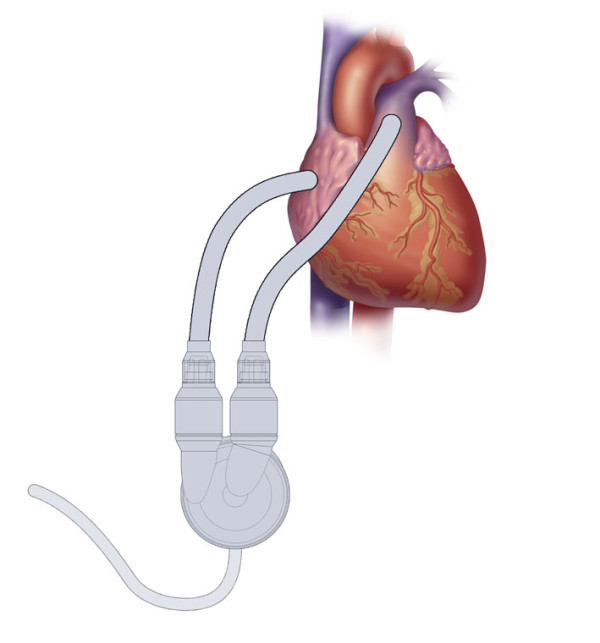
**Abiomed AB5000 RVAD™**.

The use of a ventricular assist device (VAD) for cardiogenic shock is well known and a variety of conditions--both medical and surgical--have warranted their use [11]. The general criteria for their implantation are persistent hemodynamic instability despite maximal pharmacological measures, often including the use of an IABP [12]. The types of VADs vary depending upon whether they are used as (a) a temporary unit with eventual removal following myocardial recovery,(b) a long-term unit with the goal of removal upon successful cardiac transplantation, or (c) a permanent unit in cases of Destination Therapy (DT). In this case and others, the VADs most commonly used in the setting of acute cardiogenic shock are extra- or paracorporeal devices--the intent being short-term (i.e. days to weeks) support with eventual removal upon myocardial recovery. The Abiomed AB5000™ left VAD (LVAD) is a paracorporeal unit with an inflow attachment from the left atrium or left ventricle and an outflow graft to the ascending aorta. There is a right VAD (RVAD) counterpart with inflow from the right atrium or right ventricle and outflow to the main pulmonary artery (Figure 2). In either case, the VAD bloodpump is connected to a fully automated console and can deliver up to 7 liters per minute of blood flow in a pulsatile form. The AB5000™ is FDA-approved and available worldwide, residing in both transplant and non-transplant centers. Among the advantages of the AB5000™ is its versatility: it can be used as a right sided or left sided VAD and can provide days, weeks, or months of support. As such, it can sustain the circulation for short or intermediate stages of native heart recovery or serve as a (off-label) bridge-to-transplant device. In this particular case, short-term application was all that was needed.

The circumstances of this case are profound and not completely understood. For example, the usual cardiovascular toxicities of loco-regional anesthesia with bupivicaine are bradyarrhythmia and hypotension. In this case, the initial reaction was tachycardia and hypertension, suggesting a possible systemic reaction to the epinephrine with inadvertent intravascular administration. The subsequent events, however, were equally confusing--beta-blocker use followed by ventricular fibrillation, hypotension, and pulmonary edema requiring ACLS. The cardiac dysfunction was global and persistent and was not a structural problem such as occult coronary arterial, valvular, or congenital disease. Rather, it appeared to be a profound chemical reaction that was not immediately reversible. In previously reported cases of local anesthetic induced cardiovascular collapse, the successful use of an intravenous lipid infusion has been described [9,10]. However, these clinical reports presume a bupivacaine based toxicity, which may or may not have been the case reported here.

**Figure 3:**
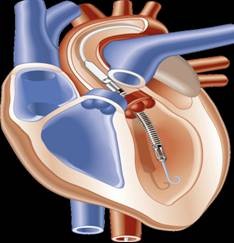
**Abiomed Impella™**.

Although the exact etiology and mechanism of the cardiogenic shock may not be elucidated, the treatment is noteworthy. As previously mentioned, the role of VADs is in the setting of refractory cardiac failure and advanced mechanical support devices have salvaged medical and surgical conditions in an ever increasing number of scenarios. The use of the Abiomed AB5000™ was simply based on its availability. Other devices would certainly have been considered, including an extra-corporeal membrane circuit with an oxygenator (ECMO) or the newly FDA-approved micro-axial flow Impella™ (Abiomed, Inc., Danvers, MA) pump (Figure 3). The Impella ™ pump would have been an attractive device in this case report since it can be placed either percutaneously or by direct femoral artery cutdown in the case of the 2.5 L/min or 5.0 L/min versions respectively. However, this device was not available or FDA-approved at that time.

In summary, the use of a VAD in the setting of a loco-regional anesthesia induced profound cardiogenic shock should be considered. Rapid transfer from an outpatient setting to a facility equipped with some form of advanced mechanical circulatory support device can translate into lives saved.

## Competing interests

LES discloses a financial relationship with Abiomed, Inc., serving as a speaker and consultant. EC and CD have no competing interests.

## Authors' contributions

LES, EC, and CD were responsible for the preparation and accuracy of the manuscript. All authors read and approved the final manuscript.
